# Mapping Antennal Sensilla of Boxelder Bugs (*Boisea trivittata*) as the First Step in Understanding Overwintering Aggregation Behavior

**DOI:** 10.3390/insects17010006

**Published:** 2025-12-20

**Authors:** Anika Sharma, Stephen A. Kells

**Affiliations:** Department of Entomology, College of Food Agriculture and Natural Resource Sciences, University of Minnesota, St. Paul, MN 55108, USA; anikasharma9125@gmail.com

**Keywords:** functional structures, Hemiptera, morphology, olfaction, peg-in-pit sensillum, SEM, ultrastructure

## Abstract

Understanding the overwintering behavior of boxelder bugs (*Boisea trivittata*) is critical for preventing initial aggregation and secondary movement inside structures. Like many Hemiptera, boxelder bugs exhibit behaviors driven by allomones, including host cues, and field observations suggest that alarm pheromones and chemical cues mediate their end-of-season aggregation. We mapped the external morphology, ultrastructure, and distribution of the antennal sensilla of overwintering boxelder bugs via scanning electron microscopy to understand the placement of antennal sensory structures. Cuticle morphology SEM identified 20 subtypes distributed across seven categories of sensilla, namely, sensilla trichodea, sensilla basiconica, sensilla chaetica, sensilla campaniformia, sensilla coeloconica, sensilla ampullacea, and sensilla bell-mouthed, though the distribution varied considerably depending on the antennal segment. The majority of sensilla on the scape, pedicel, and basiflagellomere were mechanoreceptors, with the distiflagellomere supporting a considerable number of chemoreceptors for olfaction. However, two patches of sensilla coeloconica were located distally on the outer lateral pedicel and basiflagellomere. Mapping the number, location, and distribution of sensilla present on their antennae enables further investigations of allomone-mediated seasonal aggregation, resulting in the development of more effective pest prevention strategies against this insect.

## Simple Summary

Insects use sensory structures called sensilla to sense the world around them, for example, to smell, detect the temperature and humidity, or position their limbs. Understanding the placement and distribution of these sensilla will enable further studies to advance prevention and control measures against insect pests. Boxelder bugs are free-living herbivores in the landscape; however, during the fall season, they exhibit behaviors to avoid adverse conditions, resulting in these insects entering buildings, such as residences, industrial structures, and commercial facilities. The majority of scent receptors on boxelder bugs are localized on their last antennal segment, with a few potential patches on the second and third segments. The majority of the receptors on segments closer to the head detect physical and mechanical changes. Knowledge of the locations of these sensory structures will enable further understanding of these insects and is necessary for studying their behaviors that result in their aggregation within structures, enabling improved methods of pest prevention and control.

## 1. Introduction

The eastern boxelder bug, *Boisea trivittata* (Say) (Heteroptera: Rhopalidae), is native to North America and a specialist herbivore of the boxelder tree, *Acer negundo* L. (Aceraceae) [1], and other plants [2]. With increasing temperatures during springtime, adults emerge from overwintering harborages to initiate feeding and begin their reproductive season, which lasts through the summer [3]. In late summer or early fall, both nymphs and adults cease feeding and depart from host trees [4]. In late fall and in preparation for winter survival, adult *B. trivittata* aggregate in the natural cracks and crevices of rock piles, tree trunks, and similar sites with strong sun exposure. In anthropogenic structures, boxelder bugs mainly aggregate on the outside of south- or west-facing surfaces and move into buildings, including homes, commercial and industrial buildings, and other structures.

Boxelder bugs are generally not considered pests while in the landscape. However, their status as a pest changes when they start to aggregate in buildings, clustering under the exterior structural layers, such as exterior vinyl or wood siding, metal cladding, or brick veneer. Depending on the gaps provided in the exterior infrastructure, with repeated diurnal heating and cooling of building surfaces, boxelder bugs may subsequently move deeper into building structures. This subsequent movement results in boxelder bugs entering living spaces, mainly becoming an aesthetic pest for people adverse to insect activity in their homes.

When boxelder bugs enter operational areas of commercial or industrial sites, they can create serious economic damage. As economic pests, their aggregation behavior and subsequent movement may contaminate food or pharmaceutical products, or damage electrical and other equipment. Such damage from boxelder bugs is seldom reported publicly, but citations of other species with similar behaviors demonstrate the potential extent of damage. For example, *Halyomorpha halys* adults also aggregate inside anthropogenic structures to overwinter [5,6], and several car-carrier vessels from South Korea were temporarily refused entry into Australia because of the detection of overwintering *H. halys* in the vessels and cars [7]. This caused significant economic losses, as stink bugs delayed 10,000 vehicles following increased biosecurity checks. Understanding this aggregation behavior is the first step in predicting, preventing, or reducing the use of structures as overwintering sites and avoiding economic losses.

The aggregation of adults to the host tree is mediated by semiochemicals released by the boxelder tree [1]. Similarly, the aggregation of adults away from the host tree is believed to be mediated by pheromones [8]. Boxelder bug antennae consist of four segments: the scape, the pedicel, and two flagellomeres. The different sensilla present on all these segments play a significant role in habitat selection, aggregation behavior, host finding, courtship, and mating behaviors [9]. With their potential to cause damage from overwintering behaviors, it is necessary to investigate the antennae of these insects to understand possible locations for further electrophysiology recording used to support behavioral studies. Previous studies have demonstrated that insects detect semiochemicals through olfactory receptor neurons (ORNs) enclosed in olfactory organs [10,11]. For example, *Cimex lectularius* L. detects semiochemicals through ORNs in the olfactory sensilla on their antennae [12,13]. A study conducted by Olson et al. demonstrated that olfaction sensilla related to a patch on the distal pedicel mediated off-host aggregation behavior [14]. Such a specialty patch was not part of the regular areas of olfaction on the flagellomeres of this insect, and traditional electroantennography from whole antennae was very difficult with bed bugs.

To understand the placement and number of sensilla in preparation for further pheromone work, we performed a morphological study of boxelder bug antennae via scanning electron microscopy. The aim of the present study was to understand the morphology, location, and distribution of the antennal sensilla. This work is a prerequisite for future electrophysiological and behavioral studies of the antennal sensory system involved in intraspecific chemical communication, with the aim of eventually determining an effective way to control or reduce overwintering aggregations of boxelder bugs in human-made structures.

## 2. Materials and Methods

### 2.1. Insect Collection and Sample Preparation

Aggregating adult boxelder bugs were mass collected from boxelder trees (*Acer negundo* L.) and nearby buildings around the St. Paul Campus, University of Minnesota (Falcon Heights, MN, USA), and from a residence in Lino Lakes, MN from September to November over three years (2022–2024). The collected specimens were preserved in 70% ethanol (Decon Labs, Inc., King of Prussia, PA, USA) until required for morphological examination. Whole heads with intact antennae were excised from the preserved samples for scanning electron microscopy.

The excised heads of the adult boxelder bugs were transferred to vials containing 70% ethanol and sealed afterwards, with one sample/vial. The samples were sonicated for 2 min to remove foreign particulates, and then they were dehydrated over four days to stabilize soft tissues in a graded alcohol series of 70%, 80%, and 100% ethanol. An initial survey of the SEM micrograph images of the collected samples preliminarily confirmed specimen handling techniques, and the best images with a lack of damage were selected.

### 2.2. Scanning Electron Microscopy (SEM)

The excised heads of the boxelder bugs (*n* = 12), both males and females, were individually mounted either dorsally or ventrally on aluminum stubs (AMRAY 1000/1200, aluminum, 15 mm pin height; Electron Microscopy Sciences, Hatfield, PA, USA) with double-sided carbon conductive tape (PELCO Tabs™, Carbon Conductive Tabs, 25 mm dia) and sputter-coated with 60/40 gold–palladium with a 2–3 nanometer thickness (Cressington 108 Auto; Ted Pella, Inc., Redding, CA, USA). The initial SEM micrographs were obtained using a Hitachi SEM model S3500N (Hitachi High Technologies Corp., Tokyo, Japan), and the final high-resolution images were obtained with an accelerating voltage of 10 kV to 20 kV via a Thermo Fisher Apreo 2S SEM (Thermo Fisher, Waltham, MA, USA).

### 2.3. Sensilla Mapping and Enumeration

The identification of sensilla and examination of their features were based on Nowinska and Brozek [15,16], as well as a morphological comparison of the antennal sensilla of different species of Heteroptera characterized in studies conducted by Silva et al. [17], Gonzaga-Segura et al. [18], and Taszakowski et al. [19]. Micrographs of each antennal segment, including the scape, pedicel, basiflagellomere, and distiflagellomere, were taken to enumerate the different types of sensilla. This enumeration was carried out by counting the sensilla in 100 μm^2^ sections from the dorsal and ventral surfaces of each segment, which enabled the inclusion of sensilla on the lateral surfaces in these estimates. The type of sensilla, their placement, and their density were tabulated with a representative image.

## 3. Results

### 3.1. Gross Morphology of Antennae

**Figure 1:**
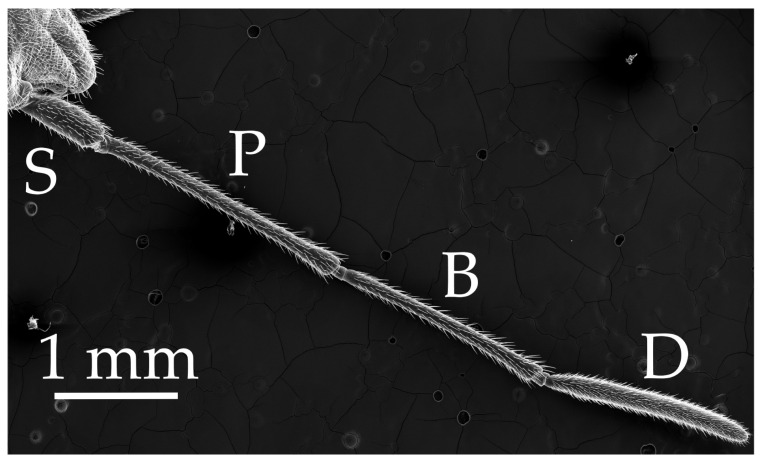
Whole antenna of boxelder bug presented dorsally, indicating scale and segments, including scape (S), pedicel (P), basiflagellomere (B), and distiflagellomere (D). The basiflagellomere and distiflagellomere are synonymous with flagellomeres 1 and 2, respectively.

Boxelder bug antennae consist of four antennomeres, including the scape (883.6 µm), pedicel (2.2 mm), basiflagellomere (1.8 mm), and distiflagellomere (1.7 mm; Figure 1). The scape is a short and thick segment. The pedicel is the longest segment, whereas the basiflagellomere and distiflagellomere are subequal in size. The density of sensilla increased from the first to the last segment, with the distiflagellomeres (fourth segment) having the greatest density of sensilla. Sexual dimorphism was not apparent in either antenna morphological or sensilla characteristics.

### 3.2. Sensilla Types and Arrangement

**Figure 2:**
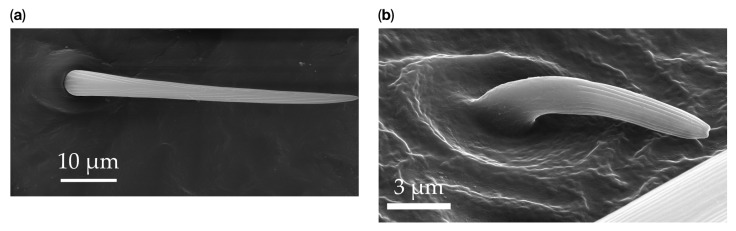
Example of sensilla inserted into (**a**) a flexible socket versus (**b**) an inflexible socket.

The principal system for describing the type of sensilla in *B. trivittata* is based on determining whether they have a porous or nonporous surface and the type of connection with the cuticle (i.e., a flexible or inflexible socket) (Figure 2). Then, the sensilla can be differentiated on the basis of size, shape, the presence or absence of pores, and the presence of a grooved or smooth surface. Seven types of sensilla, subdivided morphologically, were identified in the boxelder bug specimens: sensilla trichoidea (ST), sensilla chaetica (SCh), sensilla basiconica (SB), sensilla coeloconica (SCo), sensilla campaniformia (SCa), sensilla ampullacea (SA), and sensilla bell-mouthed (SBm).

**Figure 3:**
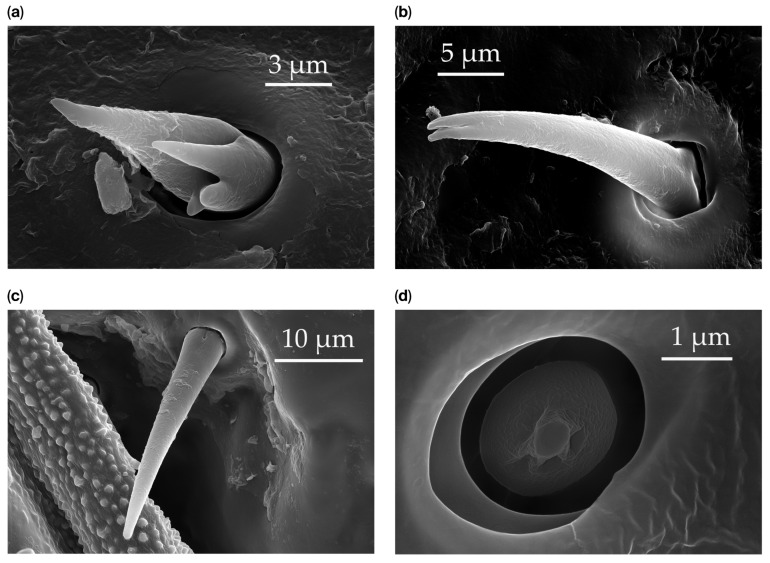
Modifications and different views of various sensilla. Three different modifications occurred with sensilla basiconica 1 (**a**–**c**) compared with SB1 (Table 3). A top-view of SCo1 showing an additional placement in the cuticular pore (**d**).

Generally, flexible sensilla emerge from cuticular sockets, whereas inflexible sensilla appear contiguous with the cuticle (Figure 2). Aporous sensilla can have either flexible or inflexible sockets, whereas porous sensilla always arise from inflexible sockets. Sensilla *trichoidea* can be distinguished as flexible hair-like structures (Table 1). Sensilla chaetica are long thick hairs (Table 2). Sensilla basiconica are cone-shaped with a porous or aporous surface (Table 3). Sensilla coeloconica are short peg-like structures embedded in a pit (Table 4). Sensilla campaniformia are flat, oval structures on the surface of the cuticle (Table 5). Sensilla ampullacea consist of open pores on the cuticle surface, while sensilla bell-mouthed have a characteristic pocket on the surface (Table 5). Some modifications of sensilla occur, though the modified sensilla are not differentiated enough to be considered to belong to additional subclasses (Figure 3).

## 4. Discussion

Boxelder bugs have a distribution of sensilla consistent with biological function, and understanding this distribution will enable the identification of regions of focus for further electrophysiological studies. In *C. lectularius*, electroantennography related to the aggregation pheromone was best optimized when a patch was located on the outer lateral distal pedicel [14]. This patch on bed bugs was located after considerable work involving progressively removing structures and ultimately stopping the aggregation pheromone response. Further, the work indicated an arrestant rather than an attractant behavior in response to the aggregation pheromone [23]. This present survey of the sensilla on boxelder bugs provides the placement of key receptors along all four segments without having to assume that chemoreception is solely isolated to the distal two flagellomeres. Further, interferences in signals from unrelated receptors can be minimized during electroantennography through the placement of different electrodes closer to the sensilla of interest. Given the short period during which boxelder bugs exhibit seasonal aggregation and the potential for additional procedures to determine behavioral response, such knowledge is important for deciding the next steps in evaluating the aggregation pheromones used by boxelder bugs.

The general function of sensilla on boxelder bug antennae can be surmised based on their morphology and distribution. Porous sensilla are related to olfaction, whereas mechano-, thermo-, and hygro-receptors are often associated with aporous sensilla [19]. The type of cuticular attachment—a flexible or inflexible socket—serves as an important morphological indicator of sensillum function. Sensilla with inflexible sockets are generally associated with chemosensory roles, as neuro-dendrites are inserted inside the sensillar shaft. Mechanoreceptors generally have a flexible attachment with the cuticle, possessing a membrane that enables movement at the base. Visible porosity, or a lack thereof, provides indications of chemo- versus mechano-reception. Chemoreceptors can be multiporous olfactory sensilla or uniporous gustatory sensilla, whereas mechanoreceptors and thermo-hygroreceptors are attributed to aporous sensilla [24,25,26,27,28]. The most common mechanoreceptors are sensilla trichodea, sensilla chaetica, and sensilla campaniformia [24,28,29]. Chemoreceptors consist of one or more sensory neurons responsible for gustation and olfaction. Common chemoreceptors are sensilla basiconica, sensilla coeloconica, and sensilla trichodea [28,30]. In boxelder bugs, there are several exceptions to these general characteristics; specifically, some sensilla have flexible sockets and at least one pore (e.g., ST1 and SB1), and some have no pores but appear to be inflexible (ST5) (Table 1 and Table 3). Such exceptions are noted for future work. Sensilla are distributed across the antenna and can be summarized by antennal segment.

### 4.1. Scape

The scapes of boxelder bugs have numerous sensilla types, though at a lower density than other segments. The scape has sensilla trichoidea (ST1, ST2, ST3, and ST4), sensilla chaetica (SCh1, SCh2, SCh3, and SCh4), sensilla basiconica (SB1), sensilla ampulacea (SA), and sensilla campaniformia (SCa), which are aporous sensilla embedded in a flexible socket. In boxelder bugs, based on the morphological study, it can be stated that sensilla trichoidea, sensilla chaetica, sensilla campaniformia (SCa), and sensilla basiconica (SB1) are responsible for mechanoreception (Table 1, Table 2, Table 3 and Table 5). Sensilla trichoidea and chaetica are the dominant mechanoreceptors in *B. trivittata* and numerous other insect groups [14,15,18,31]. Sensilla trichoidea are considered the most common mechanoreceptors, and, depending on their location on the antennomere, they act as either exteroceptors or proprioceptors [32,33].

In *B. trivittata*, the proprioceptive sensilla (SB1), located within the joints at the proximal end of the scape and at the articulation between the scape and pedicel, function to detect the positional orientation of the antenna [18]. These sensilla are often recognized as short trichoidea, chaetica, or basiconica. An investigation of various heteropteran species, including those in the infra-orders Cimicomorpha, Gerromorpha, Nepomorpha, and Pentatomomorpha, characterized these sensilla as mechanoreceptive sensilla basiconica [15,34,35,36]. In *Leptoglossus zonatus*, small sensilla present between the scape and pedicel were identified as small, smooth sensilla trichoidea [18], which is in contrast to our finding of SB1 at these sites.

Sensilla campaniformia (SCa, Table 5) are strain sensors/detectors that monitor mechanical deformations of the cuticle and provide mechanical stimulation. These sensilla are predominantly localized near articulations [37]. In *B. trivittata*, a cluster of SCa was identified, exhibiting morphological features consistent with campaniform sensilla in *Leptoglossus* [19]. These sensilla are located proximally on the dorsal side of the scape and are also distributed sparsely across other antennomeres. Similarly, in *Leptoglossus*, a group of dome-shaped, aporous sensilla, named the A4 type, were documented on the ventral side of the scape by Taszakowski et al. [19], and these are morphologically analogous to mechanosensilla SCa. In addition to their presence on the antennae, SCa are extensively distributed on legs, including the trochanter, femur, tibia, and tarsal segments [38,39,40,41]. These sensilla occur as both isolated individual units and organized clusters, often positioned in regions subject to significant mechanical stress, such as near articulations or on cuticular surfaces that undergo frequent deformation during locomotion.

### 4.2. Pedicel

**Figure 4:**
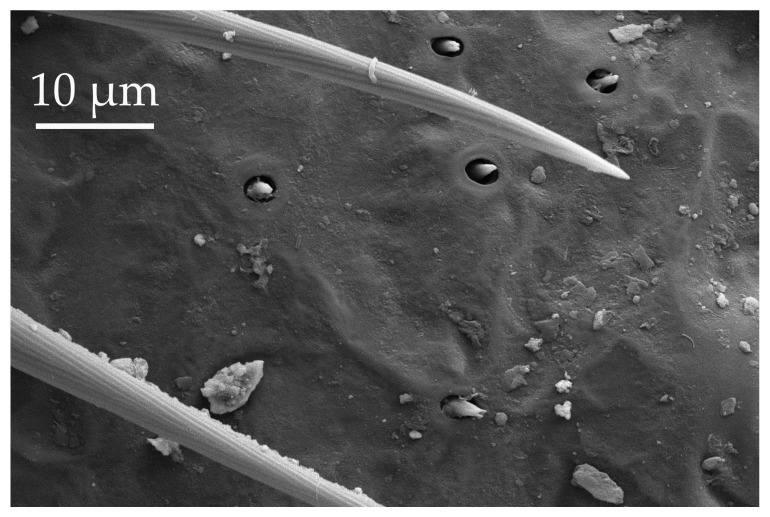
A group of five SCo2 located on the outer lateral surface of the pedicel and basiflagellum.

The pedicel of boxelder bugs has sensilla trichoidea (ST1, ST2, and ST3), sensilla chaetica (SCh1 and SCh2), sensilla basiconica (SB1), sensilla coeloconica (SCo2), sensilla ampulacea (SA), and sensilla campaniformia (SCa) (Table 1, Table 2, Table 3, Table 4 and Table 5). Sensilla trichoidea are the most numerous, with the other sensilla types scattered and interspersed along the segment. Sensilla coeloconica (SCo2) occur in a patch at the distal end of the outer lateral pedicel and basiflagellomere (Figure 4). Both sensilla coeloconica (SCo2) and sensilla ampulacea (SA) are assumed to be thermo-hygroreceptors. Their role is thought to support behaviors that prevent water loss and provide protection against temperature fluctuation. Kleineidam and Tautz [42] suggested two possibilities regarding the morphological structure of SCo and SA: both are embedded below the cuticle surface to either save space on the surface of the antennae or protect the sensory pegs against environmental extremes. However, with the placement of SCo2 in a latero-distal patch, there may be additional functions, given the placement of a similar patch on the pedicel of *C. lectularius* [14]. This patch is noted for further behavioral studies. The numbers of SCa gradually decrease in the distal segments.

### 4.3. Flagellomeres

Along the main segment, the basiflagellomere consists mainly of sensilla chaetica (SCh1) mechanoreceptors and sparsely interspersed sensilla campaniformia. Similar to the pedicel, there is a cluster of SCo2 on the outer lateral side, towards the terminus of this segment. The presence of these SCo2 should be investigated further to determine whether there are any differences between the patches on these two segments. The presence of numerous mechanoreceptors, and the incidental stimulation thereof, presents an additional risk of extra signal noise between the sites of olfaction and the placement of different and indifferent probes. Future studies will have to consider or accommodate these degraded signal-to-noise ratios.

The distiflagellomere of the boxelder bug shows specialization towards olfaction, as this terminal segment has a distinct diversity of sensilla with one or more pores, including SB2, SB3, SB3a, SB4, SB5, and SCo1 (Table 3 and Table 4). Chapman [20] stated that greater numbers of chemoreceptors increase the possibility of perception of the chemical environment. Still, there are mechanoreceptors on this last antennal segment likewise showing diversity in length and conformation, such as ST3, ST4, ST5, SCh1, SCh4, and SCa (Table 1, Table 2 and Table 5).

In this study, we classified porous sensilla into several forms: SB2, SB3, SB3a, SB4, and SB5. However, based on Steinbrecht [43] and Altner and Prillinger [44], the porous system and other morphological features differ in two (SB2-SB3a and SB4-SB5) distinguished groups. The first group consists of SB2, SB3, and SB3a, and they possess a non-grooved wall densely covered with clearly visible multiple pores along the surface. The other sensilla group (SB4 and SB5) has a porous wall that is grooved. The SB4 group is represented by deep longitudinal grooves and a single terminal pore. In *B. trivittata*, SB5 have shallow grooves consisting of linearly arranged pores. Sensilla basiconica in our study belong to both “single-walled wall pore” (SB2, SB3, SB3a, and SB5) and “double-walled wall pore” classes (SB4) [41,44].

Morphologically, chemosensitive sensilla basiconica are well represented in *B. trivittata*. Sensilla with olfactory functions are confined to the distiflagellum, suggesting that the last segment is a primary “olfactory segment.” Evidence of the terminal segment as a principal site for olfaction has also been reported in other heteropteran species. A study conducted by Andersen and Ball [45] reported more nervous tissue in the fourth segment of *Oncopeltus* fasciatus than in the remaining three segments. *Oxycarenus laetus* Kirby (Hemiptera) also reported a high sensilla density on the last segment compared to the other segments [46].

## 5. Conclusions

This survey was undertaken to map the various sensilla of boxelder bugs, and the identification of the distiflagellomere as the principal site of olfaction helps to focus our electrophysiology work on this site. The presence of two SCo2 patches on the latero-distal pedicel and basiflagellomere suggests that extra work should be undertaken to determine whether seasonal aggregation behavior would still occur after removing the distiflagellomere. The presence of many mechanical sensilla and the distance from the site of neuro-activity suggest that signal interference may occur, and focusing specifically on sites will reduce unnecessary noise. This work greatly enhances our ability to conduct further studies within the short period during which boxelder bugs display their seasonal aggregation behavior. By understanding the seasonal aggregation behavior of this insect, we can examine ways of preventing their occurrence in human-made structures when they go from being a free-living insect to a structural pest in residences, as well as at commercial and industrial sites.

## Figures and Tables

**Table 1:** Sensilla trichoidea (ST) are flexible hair-like structures, and the group includes the longest type of sensilla on boxelder bugs. Depending on the subtype, they are generally located on all four segments.

Subtype	Description and Location	Representative Image
ST1	ST1 are short, straight, cone-like sensilla, with a flexible socket and a smooth surface lacking grooves, terminating with an apical pore. These sensilla are found only on the distal end of the scape and pedicel at a density of less than 5 sensilla per 100 μm^2^.	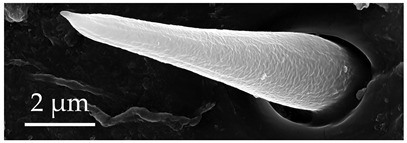
ST2	ST2 are short, thin hair-like sensilla, with a base inserted in a flexible socket, grooves along the surface, and an aporous, pointed tip. ST2 protrude at an angle to the surface, directed distally. ST2 are singularly scattered on all the antennomeres, except for the distiflagellomere, at a density of less than 5 sensilla per 100 μm^2^.	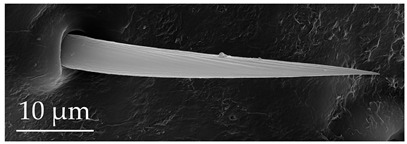
ST3	ST3 are straight hairs with a flexible socket, ribbed surface, and an aporous, blunt tip. The ribs extend from the base to the tip of the sensilla. Mid-shaft, the diameter of this sensillum stem is subequal. ST3 are larger than ST2 and present on all antennomeres at a density of 5–7 sensilla per 100 μm^2^.	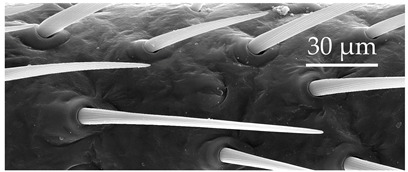
ST4	ST4 are long, hair-like sensilla, embedded in a flexible socket, bearing a ribbed surface that starts above the base and ends before the tip. These sensilla are aporous. The shaft diameter uniformly tapers along the whole length, with a long thin pointed tip. They are found on the scape and distiflagellomere at a density of less than 5 sensilla per 100 μm^2^.	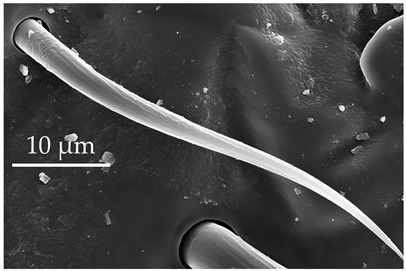
ST5	ST5 are long, straight or slightly curved, thin hair-like structures, arising from an inflexible socket. The sensilla wall is smooth with no pores or grooves on the surface. ST5 are uniformly distributed exclusively on the distiflagellomere at densities of 9–12 sensilla per 100 μm^2^.	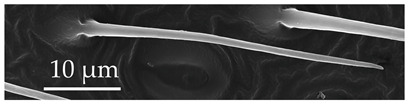

**Table 2:** Sensilla chaetica (SCh) are thicker than sensilla trichoidea. They are found on all antennomeres and consist of a relatively stout shaft arising from a flexible socket. Their outer surface is sculptured by deep longitudinal grooves or pronounced ribs. Several subtypes are identified according to shaft size, location and branching; all subtypes explained below are aporous and assumed to be mechanoreceptors.

Subtype	Description	Representative Image
SCh1	In addition to the general description of the group, SCh1 subtypes are of intermediate length. SCh1 are slightly curved and become parallel with the surface oriented distally. SCh1 are evenly distributed on all antennomeres at densities of 6–9 sensilla per 100 μm^2^.	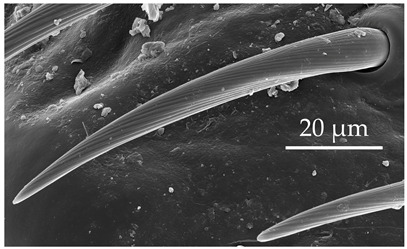
SCh2	SCh2 are long sensilla with a rounded tip. SCh2 are scattered around the circumference of the scape and pedicel segments at a density of less than 5 sensilla per 100 μm^2^.	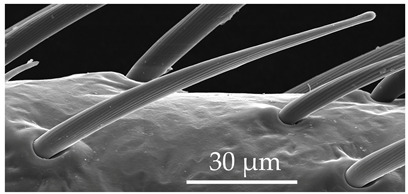
SCh3	SCh3 are short sensilla. Proportional to length, they are thicker than SCh1 and SCh2 and branched at the tip. SCh3 are sparse, with a density of less than 5 sensilla per 100 μm^2^, and are found on the scape only.	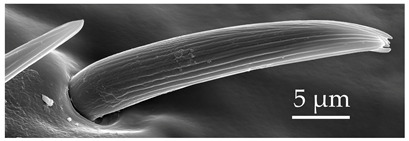
SCh4	SCh4 are short, curved, and distributed on the scape and distiflagellomere at a density of less than 5 sensilla per 100 μm^2^.	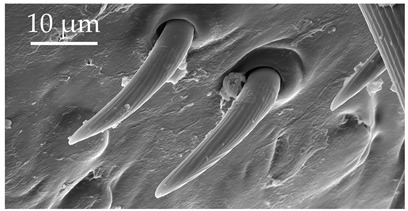

**Table 3:** Sensilla basiconica (SB) are porous or aporous sensilla, arising from flexible or inflexible sockets depending on the subtype.

Subtype	Description	Representative Image
SB1	SB1 have a flexible socket with a pore at the base. These are branched or unbranched cones (Figure 3a,b). The distal stem of SB1 is stiff and blunt. SB1 are found individually near the proximal end of the scape and pedicel (Figure 3c) at a density of less than 5 sensilla per 100 μm^2^.	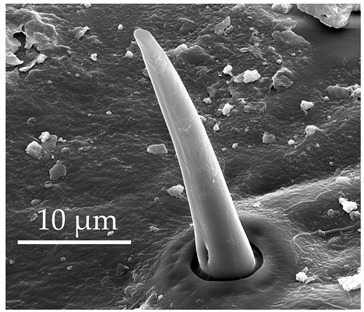
SB2	SB2 have an inflexible socket and a distinctly porous surface. The sensillum stem is wide and stiff, with several gland pores at the base. Numerous raised ellipsoid shaped pores are present over the entire surface. SB3 are very few and occur on the distiflagellomere at a density of less than 5 sensilla per 100 μm^2^.	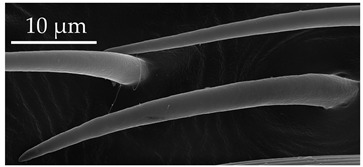
SB3	SB3 are cone-like structures that arise from an inflexible socket, with a proportionately thick stem and a pointed tip. They are either straight or bent towards the distal end of the antenna. There are 8 to 10 large pores at the base. Numerous comma-shaped nano-pores are distributed over the entire sensillum surface. These sensilla are relatively numerous on the distiflagellum at densities of 5–8 sensilla per 100 μm^2^.	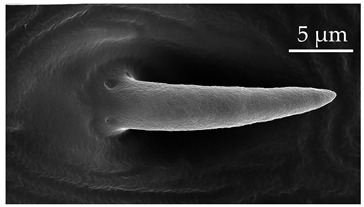
SB3a	SB3a are similar to SB3 but distinctly shorter. They are cone-shaped, slightly curved, asymmetrical-bulbous structures with pores on the entire surface. Around the sensillum base, there are several gland pores. SB3 are present only on the distiflagellomere and few in number, with a density of less than 5 sensilla per 100 μm^2^.	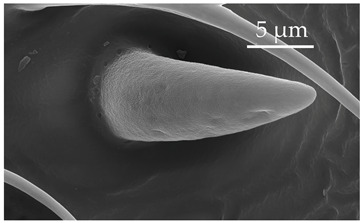
SB4	SB4 are very short cones embedded in an inflexible socket, with a distal end tapering to a pore. Proximal to the base, these sensilla are smooth, with deep longitudinal grooves occurring on the mid-shaft and extending to the end. Most of these sensilla are found to be strongly curved and oriented distally. SB4 are numerous but scattered on the distiflagellomere at densities of 9–10 sensilla per 100 μm^2^.	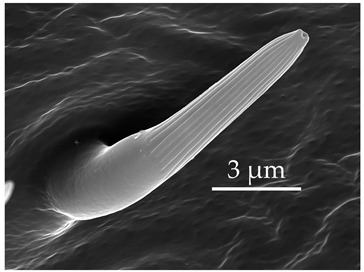
SB5	Of the SB group, SB5 are long sensilla. SB5 have an inflexible socket, with longitudinal shallow grooves present along the entire sensillum. Within these grooves, there are linearly arranged pores. SB5 occur exclusively on the distiflagellomeres at a density of less than 5 sensilla per 100 μm^2^.	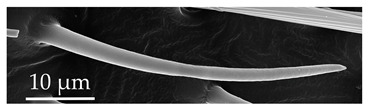

**Table 4:** Sensilla coeloconica (SCo) are peg-in-pit sensilla with a sensory structure sunk into a cavity. They generally have smooth surfaces that are deep within the cavity. They are embedded in an inflexible socket. Compared to the abundance of other sensilla, sensilla coeloconica are few in number and often appear singularly [20,21,22].

Subtype	Description	Representative Image
SCo1	SCo1 are short pegs inserted deeply in the pit. The distal end of the sensilla tapers into a relatively wide opening. These sensilla are not numerous and are found exclusively on the dorsal surface of the distiflagellomere at a density of less than 5 sensilla per 100 μm^2^.	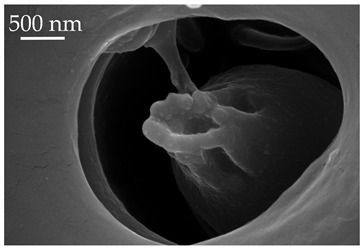
SCo2	SCo2 protrude from a sensillum pit. The distal end of these sensilla taper into a terminal pore similar to SCo1, but the pores tend to be smaller in diameter. These sensilla are located in a grouping of five sensilla, laterally on the caudal or outer-facing surfaces of the distal pedicel and basiflagellomere (Figure 3). These sensilla are consistent in number, but their arrangement/pattern varies among individuals.	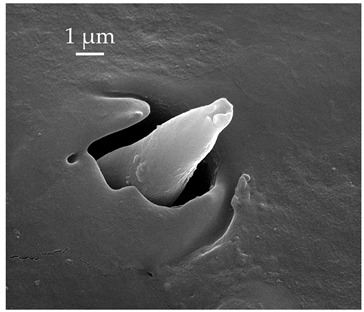
	Note: the pore to the left of SCo2 is a *Sensillum ampullaceum* (SA).	

**Table 5:** Miscellaneous sensilla found, including sensilla campaniformia (SCa), sensilla ampullacea (SA), and *sensilla bell-mouthed* (SBm).

Subtype	Description	Representative Image
SCa *	SCa are flat, oval, and cupola-shaped sensilla with flexible sockets and a single central pore. SCa occur in groups of 4–5 located at the base of the scape, but they also occur singly across all segments, usually in areas more susceptible to stress, at densities of less than 5 sensilla per 100 μm^2^.	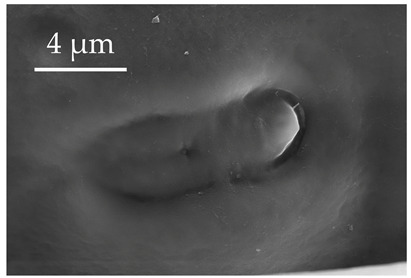
SA	These are small pegs embedded in a small tube, with only a round opening visible from the surface. In *B. trivittata,* they are found scattered on the scape, pedicel, and basiflagellomere at densities of less than 5 sensilla per 100 μm^2^.	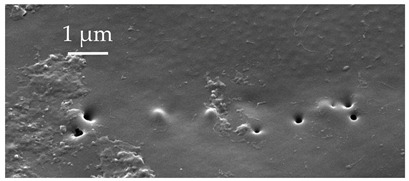
SBm	SBm are funnel-shaped pits with multiple cuticular folds. Only one sensillum is present on the articulated joint connecting the pedicel and the basiflagellomere.	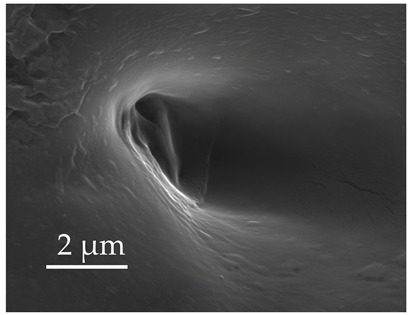

* Image brightness adjusted by +30.6%.

## Data Availability

The original contributions presented in this study are included in the article. Further inquiries can be directed to the corresponding author.

## Abbreviations

The following abbreviations are used in this manuscript:

DOAJ	Directory of Open-Access Journals
SA	Sensilla ampullacea
SB	Sensilla basiconica
SBm	Sensilla bell-mouthed
SCa	Sensilla campaniformia
SCh	Sensilla chaetica
SCo	Sensilla coeloconica
ST	Sensilla trichoidea
SEM	Scanning electron microscope
